# Emerging Infectious Diseases and Disease Emergence: critical, ontological and epistemological approaches1

**DOI:** 10.1086/726979

**Published:** 2023-09

**Authors:** Matheus Alves Duarte da Silva, Jules Skotnes-Brown

**Affiliations:** University of St Andrews; University of St Andrews

## Introduction: the emergence of “emerging infectious diseases”

The COVID-19 pandemic, caused by the spread of a novel coronavirus, has once again drawn the attention of the entire globe to the threat posed by emerging infectious diseases (EIDs). In recent months, so too has monkeypox – a disease long known in West Africa – but only now in other parts of the world. What is the history of the concept of “emerging infectious diseases”? What are the critical approaches regarding the invention and circulation of this concept? And how have historians examined the appearance of “new” diseases before and after the emergence of the EID concept? This essay is intended firstly as a short, guided introduction to the recent history of this concept and to critical approaches to it. Secondly, it offers a reflective enquiry, examining how historians have discussed disease emergence. We argue that humanities scholars have critically examined the EID concept and invoked it to converse with scientists, or to comment on its contemporary realities. Simultaneously, they have demonstrated how the burgeoning interest in EIDs highlights the need for research into the ontological and epistemological factors that have posited “new” diseases and transformed them into epidemics and pandemics.

Although scientific debates on how new diseases appear can be traced to the earliest developments in biomedicine, in the years following the “Spanish” Flu Pandemic,^2^ the development of the EID concept is relatively short: it achieved mainstream traction only after the 1990s.^3^ In the previous two decades, many American and West European scientists believed that the West was witnessing a linear epidemiological transition. According to this assumption, infectious diseases had been pacified thanks to vaccination, sanitation, and antibiotics. Thus, deaths would be primarily caused by age-related problems such as cancer, degenerative, or cardiovascular diseases.^4^ This triumphant vision was partly engendered by the successful control of malaria and the eradication of smallpox. Another reason, according to Frank Snowden, was the prevailing notion of “microbial fixity.” According to this idea, future disease threats would come solely from illnesses that were already known to scientists. Coupled with this, was the idea that diseases would decline in virulence, through a process of natural selection, and would eventually come to coexist with humans.^5^ The first outbreaks of Ebola in the 1970s, the reappearance of cholera in Latin America, and plague in India, and most importantly the emergence of HIV/AIDS delivered shocks to this scientific consensus and demonstrated that infectious diseases were still a threat.^6^

To address the threat posed by such diseases, a conference was convened in 1989 by epidemiologist Stephen S. Morse and molecular biologist Joshua Lederberg. This conference, called “Emerging Viruses: The Evolution of Viruses and Viral Diseases,” was sponsored by the National Institute of Allergy and Infectious Diseases, the Fogarty International Center of the National Institutes of Health, and the Rockefeller University.^7^ The presentations and conclusions of the conference were published in 1993 under the title *Emerging Viruses*.^8^ One year later, the Institute of Medicine and National Academy of Sciences published the findings of its *Committee on Emerging Microbial Threats to Health*.^9^ Finally, in 1995, the journal *Emerging Infectious Diseases*, edited by the Centres for Disease Control, was launched.^10^ Together, these publications put together the concept of EIDs, and transformed it into a public health tool in the US. ^11^

In a foundational paper published in 1995, Morse defined EID as “infections that have newly appeared in a population, or have existed but are rapidly increasing in incidence or geographic range”—diseases like HIV/AIDS. Morse categorized other infections like cholera in South America as “re-emerging diseases,” because they “were once decreasing but are now rapidly increasing again.”^12^ Morse listed a plethora of factors which produce emerging or reemerging diseases, including: “ecological changes, such as those due to agricultural or economic development or to anomalies in climate; human demographic changes and behaviour; travel and commerce; technology and industry; microbial adaptation and change; and breakdown of public health systems.”^13^

The concept gained increased traction in the late 1990s, with the help of popular media, such as Laurie Garrett's *The Coming Plague* (1994), and the Hollywood movie *Outbreak* (1995).^14^ It has since been deployed to encompass a myriad of diseases: from once “defeated” resurging diseases such as tuberculosis in the USA,^15^ to new diseases like SARS or COVID-19, to pathogens, like anthrax, that might be used by bioterrorists.^16^ EIDs also brought renewed attention to the need for rapid international responses to emergence events. Thus, the WHO developed strategies in the 1990s to respond to outbreaks in as little as twenty-four hours.^17^ In 1997, the WHO established the GOARN (Global Outbreak Alert and Response Network), a network of 120 partners which enabled the rapid global dissemination of information.^18^

Despite such lofty, global goals, EIDs were mainly framed as threats to the West: problems “out there” that thanks to globalization could become local problems. According to Nicholas King, emerging infectious disease research gained both traction and funding by “reframing ‘international' problems in language palatable to American interests,” such as border security and terrorism.^19^ The threatening nature of such diseases ushered in a new era of what Andrew Lakoff calls “global health security.”^20^ No longer was it a question whether new diseases would be detected, but how best to predict and contain them.^21^ This framing of EIDs as global threats to the US sparked initial criticism of the concept as Americentric.^22^ However, the concept was also adapted and recreated as it circulated around the world.^23^ For instance, various African scientists have described diseases like Ebola and Lassa fever as EIDS, though they are not a problem in distant and exotic places, but are threats to the local African population. ^24^ The recent monkeypox pandemic is perhaps an ideal example of both points: a largely neglected disease known in West Africa since 1970, became an ‘emerging’ global concern after its appearance in the UK in 2022.^25^ Simultaneously, its spread has provoked alarm in other parts of the African continent, such as South Africa, where it was described as “another emergent virus.”^26^

Aside from its successes as a concept in the field of public health, broader intellectual questions posed by disease emergence have interested humanities scholars long before the 1990s. In what follows, we highlight firstly how scholars have taken the EID concept as the object of their enquiry or employed it as a critical tool. Secondly, we examine how historians have tracked the emergence of diseases through synthesizing scientific knowledge and history. Finally, we discuss some of the approaches taken within the historiography of “framed” diseases: the epistemological appearances of new diseases, or the transformation of old scourges, by emphasizing changes in science and society.

## Emerging infectious diseases: a disputed concept

The first means by which scholars have engaged with the concept of EIDs is in defining it as their object of enquiry. In this section, we analyze the critical examinations given by historians, anthropologists, and literary critics. One early example of the historical engagement with the EID concept is found in the work of medical historian Mirko Grmek. In a 1993 essay, Grmek proposed five conditions according to which a disease could be considered as emerging in the present or in the past.^27^ Grmek's categories stressed biological processes but also highlighted how social and scientific changes could make visible phenomena that had until then been ignored. This is a point that is sometimes missed by scientists.^28^

More recently, historians have used the concept of EIDs as a critical tool to intervene in current debates both in public health, and history itself. Brian Dolan's work reveals that underlying governmental models of pandemic preparedness were based on the assumption that the “next pandemic” would be a mutation of influenza. This assumption left governments unprepared, in early 2020, to deal with a coronavirus pandemic.^29^ Jon Arrizabalaga has interpreted the profusion of emerging diseases in the last few decades as proof of the risks brought by modernity. He argues that many well-known emerging diseases were created by the pharmaceutical industry – bacteria resistant to antibiotics, among others – or by the development of agriculture. To him, the pessimistic view that modernity itself is a source of disease emergence has been proven by the ongoing COVID-19 pandemic.^30^

While these historians have drawn upon the past to critique the present, a few others have demonstrated how the concept of EIDs is indebted to the past. Early- to mid-twentieth century disease ecological thinking developed in colonial settings was often overlooked in twentieth century Western public health campaigns, but took centre stage after the emergence of HIV/AIDS and Ebola.^31^ Indeed, both Joshua Lederberg and the virologist Robert Shope stressed that epidemics did not “strike societies randomly or in accord with the caprices of angry gods,” but instead reflected “relationships that human beings establish with one another and with the natural and built environments,” spreading across “fault lines created by demography, poverty, environmental degradation, warfare, mass transportation, and societal neglect.”^32^ For Warwick Anderson, such analyses signal a recognition that the developed world had “finally read the ecological lesson” provided by disease ecologists in colonial settings decades earlier.^33^ Pierre Olivier-Méthot and Bernardino Fantini's work has complemented such arguments, emphasizing that EIDs drew attention to the importance of disease ecology because they did not necessarily emerge from “particularly virulent germs,” but rather from “significant ecological changes within an ecosystem” and through “cultural and socio-economic factors” in human populations.^34^ Grmek, likewise, interpreted AIDS as “the price we pay for having radically perturbed millenary ecological equilibria.”^35^ According to Mark Honigsbaum and Olivier-Méthot, histories of disease ecology in conversation with histories of EIDs allow us both to interpret the past, and to “illuminate current scientific debates around emerging infectious diseases, and the interaction between biological, economic, and cultural factors in current pandemic emergencies.”^36^

An ecological perspective on disease can help us understand contemporary problems with EIDs. Some have even argued that EID's ecology should be brought to the forefront of environmental history. According to Linda Nash, EIDs provoke historians to investigate the relationships between environments and health. Emerging diseases, she argues, make “compelling political reasons for telling the history of disease as an environmental and social story, rather than simply as a medical and personal one.”^37^ According to Nash, from the days of Richard Preston's 1994 sensationalistic nonfiction thriller, *The Hot Zone*, emerging diseases have often been attributed to environmental degradation.^38^ Yet despite this recognition, scientific analyses of the “relevance of the environment to disease emergence” have been treated in a “very generic way.”^39^ To address this problem, historians should write environmental histories which chart the “specific social, economic, and environmental contexts that have produced the conditions conducive to outbreaks of infectious disease.”^40^

Histories of human and animal relations may be of particular importance in addressing Nash's concerns. Given the importance of animal to human spillover, human and animal relations have emerged as an important, if relatively small, subfield of EID studies.^41^ In 2003, Anne Hardy's pioneering article, “Animals, Disease, and Man: Making Connections” drew attention to the relationship between animal and human health since the mid nineteenth century, both in Europe and in European colonies. One particularly important point posed by Hardy was that human intimacies with animals have historically been identified with outbreaks of disease, often through the lens of colonial racial and class prejudices.^42^ Subsequent studies of SARS and influenza, such as those penned by Lyell Fearnley and Frédéric Keck, have continued to draw attention to how scientists have interpreted human relationships with birds, and how these have led to the positing of new diseases and their transformation into epidemics and pandemics.^43^ Similarly, Karen Brown's study of resurgent rabies in South Africa demonstrates the need to analyze human/animal relations within environmental, medical, and social history.^44^ The coronavirus pandemic has also underlined the importance of treating animals as agents of historical change on account of their epidemiological significance. Writing on the history of pangolins, Sujit Sivasundaram has argued that historically “zoonotic transfer occurs where relations between humans and animals have been unstable or where they are entering a new phase of contact.”^45^ Given climate change and the emergence of zoonotic disease, he suggests that scholars write “multi-species and even trans-species history that is about the assembly of various life forms and things generative of historical change.”^46^

While historical analyses of the emergence of the EID concept and its sociopolitical realities are relatively scarce, scholars of the medical humanities have examined this concept in more detail.^47^ Some have pointed out that despite issues with the framing of the concept, it has been “immensely successful” in gathering “international resources to fuel the development of new collaborative research…on infectious diseases” that threaten “both the North and the South.”^48^ Lorna Weir and Eric Mykhalovskiy consider the EID a concept that has been successful in altering “understandings of infectious disease in ways that mobilized widespread public health concern over new microbial threats and drove significant institutional change in the scope and form of global public health surveillance and field response,”^49^

On the other hand, the successes of this concept have simultaneously exposed its shortcomings. According to Weir and Mykhalovskiy, because the idea of disease emergence has become so popular, global-health funding bodies have prioritized attention to EIDs over endemic diseases that continue to plague parts of Africa, Asia, and Latin America.^50^ This has compounded preexisting medical funding problems within the South.^51^ Critical medical anthropologists and specialists in global health in the 1990s and 2000s have likewise drawn attention to geopolitical inequalities and EIDs. For them, emerging diseases were not simply an unavoidable consequence of globalization as their clinical definition implies. Rather, global capitalism played a critical role in the emergence of diseases such as HIV/AIDS. Since the 1990s, anthropologists have produced studies on the “structural violence” that generates vulnerability to diseases such as “poverty and economic exploitation, gender power, sexual oppression, racism and social exclusion.”^52^ To name one prominent example, in 1996, anthropologist and physician Paul Farmer critiqued the definition of EIDs, claiming that it paid insufficient attention to social inequalities, and treated emergence as a somewhat random biological event. To move beyond this problematic approach, Farmer called for a critical epistemology of emerging infectious diseases, and argued that historical, sociological, and anthropological expertise needed to be mobilized to study how inequalities of class, race, gender, and sexuality, facilitated disease emergence.^53^ Richard Parker, a medical anthropologist who was instrumental in the founding of the *Global Public Health* journal, also blamed inequalities in the IMF's programs for the breakdown of public health systems in many parts of the world.^54^ According to Méthot and Fantini, these problems persist to this day. Despite the indebtedness of the EID concept to disease ecological research, many experts still focus “on purely epidemiological and clinical aspects of emerging events” at the cost of ecological and sociological factors.^55^ Perhaps it would be better, speculate Méthot and Fantini, to refer to EIDs instead as emerging epidemics, because of “enhanced diffusion of pre-existing microorganisms thanks to several factors such as migrations, wars, travels, and trade.”^56^

Foucault-influenced anthropologists have likewise devoted attention to the concept of EIDs to explain the cosmological underpinnings of Western medical governance, and the challenges these diseases pose to biopolitics. Carlo Caduff has argued that at “the heart of the concept of emerging viruses is a particular temporality” that “naturalizes the idea of a permanent threat”. This “cosmology of mutant strains,” which emerged in the early 1980s, draws attention to the “ever-evolving nature of viruses.”^57^ Under this cosmology, medical professionals view nature as “already one step ahead”: they are perpetually “struggling to keep up with nature's relentless evolution.”^58^ Expertise is reduced to ignorance: nature produces new viruses before they are even named, and once they have been given a name, they continue to mutate *ad infinitum*.^59^ This “cosmology of mutant strains” constitutes a challenge to biopower and medical triumphalism: since pathogens are always ahead of scientists, the “bold dreams of control and eradication” must be replaced with “modest schemes of response and relief.”^60^

Along with history and anthropology, literary criticism has also provided insights into the birth of the EID concept and its sustained popularity. According to Priscilla Wald, North American publics have primarily been informed about the risks of EIDs through books, newspapers, and movies.^61^ To Wald, these stories follow a “formulaic plot”, derived from past narratives of epidemics and nineteenth-century detective novels. It commonly starts with a mysterious outbreak in an isolated place in Africa or South America. Traveling globalized networks, this faraway menace begins threatening American society. A team of doctors is then called to solve the mystery in a race against time. The heroes eventually succeed: the outbreak is contained and the disease eradicated, at least in the US.^62^ To Wald, these accounts are a tool “for making the invisible appear”: they reveal obscure biological entities and the hidden realities of globalization.^63^ Ironically, if AIDS gave a new life to the outbreak narrative, its trajectory does not follow the formulaic plot, because it was never totally contained.^64^ One questions if the ongoing COVID-19 and monkeypox pandemics will have a similar fate.

In short, critical studies of the historical, anthropological, and rhetorical aspects of the EID concept have emphasized not only biological factors, but also the social, political, and economic realities that explain the emergence of the concept, its diffusion and popularization. Such studies have, however, been largely anchored in North American and Western European experiences, paying little attention on how the concept circulated, was adapted, and reconstructed in places such as Latin-America, Africa, and South or East Asia, commonly seen by Westerners as the sources of EIDs.

## Disease emergence: an ontological question

As Stephen Morse remarked in his opening paper of the *Emerging Infections Diseases* journal, in 1995, “infectious diseases emerging throughout history have included some of the most feared plagues of the past.”^65^ This claim showcases, firstly, how history was mobilized by a generation of doctors and scientists to legitimize the EID concept.^66^ Secondly, it suggests to historians the need to track the ontological emergence of diseases.

Indeed, as remarked by historian Monica Green, every disease, from smallpox to Ebola, was at one point emerging. Historians interested in the ontological emergence of a disease must examine not only the factors that led to the original spillover event, but also those that transformed them into epidemics and pandemics, and what allowed these to persist. Advances in ancient DNA sequencing and medicine, as well as historical sciences are of use here: they allow historians to pinpoint the deep histories of diseases and to challenge established interpretations based only on historical writings.^67^ As discussed below, this approach to disease emergence has been applied by historians in two different directions: firstly, with an emphasis on the spread of diseases among human communities with little or no immunity, and secondly, with an emphasis on spillover of the disease from animals to humans.

The concept of pathocenosis, as developed by Grmek in 1969, is an important theorical milestone for a reflection on the ontological emergence of diseases.^68^ According to his definition, pathocenosis is the ensemble of diseases that existed among a population in a given time and space, which tend to stay in equilibrium. However, this equilibrium could be broken by external factors. Two examples are the arrival of black rats to Europe, which led to the Black Death, and the spread of tuberculosis, which led to the retreat of leprosy, since TB provides cross immunity against leprosy's Hansen bacillus.^69^

As remarked by Méthot, Grmek's insights anticipated some of the main arguments developed by Alfred Crosby in his essay *Virgin Soil Epidemics*, and by William McNeill in his book *Plagues and Peoples*,^70^ both published in 1976. Synthesizing historical analyses with the most advanced scientific knowledge at his disposal, Crosby stressed how the low-immunity of indigenous Americans to infectious diseases, such as smallpox, brought by European colonizers was a major reason for their demographic collapse and for European conquest.^71^ McNeill's work complemented this argument and argued that the biggest demographic disasters of humanity, such as the Black Death, the fall of the Aztec and Inca Empires, and the nineteenth century cholera waves, were the result of environmental imbalances caused by the arrival of new diseases to places with non-immunized populations. For McNeill, imperial expansion and commerce were to blame for the rupture of environmental equilibrium because they connected different environments and allowed people, animals, parasites, and micro-organisms to circulate between them. For instance, the Black Death was directly connected with the expansion of the Mongol Empire, because it bridged plague reservoirs among wild rodents in inner Asia with black rats in Europe.^72^ It is worth noting that McNeill was the only historian to participate in the 1989 conference that coined the EID concept.^73^ As he remarked in the introduction to the third edition of *Plagues and Peoples* in 1998, the spread of HIV/AIDS around the world confirmed his arguments of how disease emergence and pandemics are connected with processes of globalization.^74^

Crosby and McNeil's interpretation of the conquest of America have, however, been met with criticism, because they offer a fatalistic conclusion that indigenous American populations were fated to disappear once their pathocenotic equilibrium was broken and smallpox and other infectious disease emerged in the Americas, no matter if by violence or by peaceful contact. Their interpretation is however still potent in popular culture, as evidenced by Laurent Binet's novel *Civilizations*, which won the 2019 *Grand Prix du Roman de l'Académie Française*. Binet imagines a world where pre-Columbian populations met with Vikings adrift in the Caribbean by the year 1000, from whom they pick-up iron, horses, and smallpox, which became, one could say, part of the American pathocenosis. Therefore, in this imagined past, no demographic annihilation occurs with Columbus' arrival. Smallpox does not arrive in the Americas after 1492 because in his story it had arrived five hundred years earlier. Indigenous Americans managed then to defeat Columbus, seize his caravels, and invade and ultimately conquer Western Europe.^75^

Among historians, McNeill's argument of how the flow of people and goods between continents led to the emergence of diseases has more recently been examined in Mark Harrison's *Contagion*. By using modern knowledge on the epidemiology of diseases and archival research, Harrison emphasized the major role played by trade in the circulation of pathogens and vectors, such as rats and fleas, and showed how measures to combat them were as much driven by politics as they were by the threat posed by the disease itself.^76^ Myron Echenberg, likewise, has shown how the sea-traffic of goods, people, and also of rats and fleas between “plague ports” spread plague between 1894 and 1901 from its endemic areas in China to new locations across the globe, including South America, Australia, South Africa and the United States. Echenberg also reflected on why the disease was better controlled in some of these ports than in others. To him, the ports that focused on rat destruction (Rio de Janeiro, Buenos Aires, San Francisco), were more successful in fighting plague emergence thanthe ports of the British Empire, which relied more on disinfection.^77^ In short, Harrison and Echenberg convincingly showed how political economy and growing global capitalism were important factors in the spread of plague. Nonetheless, the price paid in these two accounts was sometimes to overshadow science's own historicity. For instance, albeit rats and fleas were framed in both books as main villains for the emergence of plague as a global scourge, these animals were not considered as such before the late nineteenth century. Even after that time, vigorous debates took place around the world on the role of rats and fleas in spreading plague and on the feasibility of killing them.^78^

John Iliffe's synthesis of the biological and social history of HIV/AIDS in Africa provides another good example of this ontological approach in action, this time focused on social factors and spillover events that transformed AIDS into a pandemic. Mobilizing up-to-date genetic studies, Iliffe situated the origins of HIV/AIDS within multiple zoonotic spillovers from simians in the 1930s-1950s, which were transformed into a silent epidemic through colonial upheavals, the migration of labor, and the inequalities of capitalism.^79^ Jacques Pépin, a former medical officer in the rural Democratic Republic of Congo, likewise showed how colonial medical interventions, “requiring the massive use of re-usable syringes and needles” helped turn HIV into an epidemic. ^80^ Most recently, William Schneider's edited volume on the *Histories of HIVs* has also demonstrated the potential for syntheses of history and biology in clarifying the emergence of diseases. He sees the history of HIV as key in understanding the future of the COVID-19 pandemic.^81^ HIV/AIDS, like COVID-19, has provided a painful lesson in how “animal viruses can transition to become epidemic or pandemic human viruses.” The fact that genetic evidence suggests that “new HIVs emerged multiple times…means they cannot be seen as random or chance events” but instead “a human made disaster.” ^82^

These ontological approaches have thus synthesized modern science and historical methods to pinpoint the appearances of pathogens in periods in which these were not yet mentioned in archives. Moreover, modern epidemiology has allowed historians to reconstruct the probable causes of their spread and understand why some places were more affected than others. Simultaneously, archival work has provided pointers as to the social, economic, and political factors that transformed diseases into epidemics and pandemics. Fascinating as such work is, it has often neglected the epistemology of disease emergence.

## Disease emergence: an epistemological problem

Infectious diseases that were emerging, either because they passed from animal to human hosts or started spreading within new communities, were often perceived differently by different peoples. Perhaps one of the richest ways in which historians have engaged with the idea of emerging diseases has been to understand the emergence, or appearance, of diseases in the past as an epistemological and social process. In other words, how and why a disease was considered a new entity followed upon transformations in science or transformations in cultural and social attitudes. A good introduction to theoretical and methodological issues in these matters can be found in Ludwik Fleck's studies on the epistemological and cultural roots of the changing nature of syphilis. Also useful is Charles Rosenberg's concept of framing disease, which stresses the scientific and social forces behind new categorizations of diseases, along with how the biological characteristics of each disease served as limiting factors to social frames.^83^ To examine the broader historiography on disease emergence as a social and scientific phenomenon, one might focus on plague and on trypanosomiasis, which is a family of diseases prevalent in sub-Saharan Africa, South Asia, and South America. These two diseases provide an excellent illustration of how social and intellectual changes can (re)invent new diseases.

Plague was reframed by microbiology between the late nineteenth and early twentieth centuries, in the first years of what was later called the “Third Plague Pandemic.” Historian Andrew Cunningham examined the transformation of plague by studying the identification of its bacillus during the Hong Kong outbreak of 1894, which started the pandemic. Cunningham argued that the French-Swiss microbiologist Alexandre Yersin and the Japanese microbiologist Shibasaburo Kitasato “had taken into their laboratories a disease whose identity was constituted by symptoms; they had emerged with a disease whose identity was constituted by its causal agent.”^84^ Therefore, Cunningham concluded, Yersin and Kitasato *transformed* plague's identity, because from 1894 onwards, a plague outbreak would be declared only after its bacillus was identified in a laboratory.

More recently, numerous historians have further developed Cunningham's argument. They have shown, for instance, that reframing plague as a microbiological entity involved more than just the identification of the bacillus. It involved the production of sera and vaccines against it, the proposing of theories about its spread, and the invention of new sanitary measures to control the bacillus and its animal “reservoirs.” Moreover, reframing plague was more of a global process than assumed by Cunningham. As we have shown in previous works, the microbiological reframing of plague involved a network of actors and laboratories in places such as Brazil, India, and South Africa. Scientific, diplomatic, and imperial forces connected them and knowledge about plague circulated between them and was transformed by them.^85^

If old diseases were reframed by microbiology, this new science, with the help of tropical medicine and indigenous medical experts, was also responsible for constructing “new” diseases, such as trypanosomiasis in Africa and in South America. African trypanosomiasis is a term that encompasses two diseases – nagana and sleeping sickness – caused by three subspecies of *Trypanosoma brucei*, a blood parasite transmitted between wildlife, livestock, and humans by the tsetse fly. Both diseases, the former in livestock and the latter in humans, cause progressive, gradual emaciation, along with neurological disturbances, followed by death. African pastoralists in numerous areas had long contended with these symptoms in livestock, and often attributed them to a disease caused by the bite of the tsetse fly. The term “nagana” itself is an Anglicization of *unakane*, an isiZulu word that has been translated as “continual pestering action,” referring to the infuriating bite of the fly.^86^ Despite trypanosomiasis's long history under a plethora of different names, numerous historians have argued that it became a problem of colonial governance in the late nineteenth to early twentieth century, during a period in which imperial powers were attempting to develop lands they had violently seized from indigenous people.^87^ African trypanosomiasis was first identified bacteriologically in the 1890s, in Zululand, and named “tsetse fly disease or nagana” by Scottish-Australian microbiologist David Bruce and his research partner and wife Mary Bruce. Later, it was studied across Africa by a host of scientists. European colonists feared that this disease would become endemic across much of the continent and even spread into other parts of their empires.^88^

Scholars such as ecologist John Ford have argued that European activities were at times responsible for the emergence and persistence of trypanosomiasis.^89^ As Maryinez Lyon notes, in her seminal study of trypanosomiasis in the Belgian Congo, it was a “colonial disease”: the political, ecological, and labor disruptions caused by colonial governance created ecological conditions conducive to the emergence of devastating trypanosomiasis epidemics.^90^ In response, historians have documented how European states attempted to prevent the disease from spreading and how Africans responded to such efforts. This involved the attempted wholesale extermination of wildlife in agricultural areas, trapping and killing tsetse flies in enormous numbers, slashing and burning vegetation in which the flies nested, the forced resettlement of Africans, and ultimately, the dusting and soaking of parts of the continent in DDT.^91^ In short, although sleeping sickness symptoms were long known in the continent, the disease “emerged” and was framed as a particular concern to colonial powers in the twentieth century due to the scramble for Africa. Colonists transformed it into a persistent series of devastating epidemics, that killed hundreds of thousands of Africans. Changes in human and animal relations were critical here. In Zululand, South Africa, for example, colonial game-protection laws coupled with the suspicion of Zulu ideas that large game was pathogenic, produced significant outbreaks of nagana, and extended its endemic area.^92^

The emergence of American trypanosomiasis, or Chagas disease, as a new nosological entity in the early twentieth century has intriguing counterpoints to its African counterpart. In 1909, while conducting work on malaria in Lassance, a village in the Brazilian state of Minas Gerais, Carlos Chagas, of the Instituto Oswaldo Cruz in Rio de Janeiro, made a breakthrough discovery. In the blood of a few people that he imagined might be infected with malaria, he found a different parasite, which he christened *Trypanosoma cruzi*. Following this identification, Chagas located the parasite's vector, known as *barbeiro* in Brazil, a common blood-sucking insect present in the hinterland of South America that nests in holes in wooden houses. Chagas identified, as a symptom of the disease, lesions in the heart, caused by the parasite, as well as related symptoms, such as fatigue and hyperthyroidism. The path followed by Chagas was unusual, not just because he started from the parasite rather than from the symptoms, but because he made a threefold discovery. Thus, as has been argued, the identification of this new disease demonstrated the Brazilian contributions to the field of microbiology and tropical medicine at the turn of the twentieth century.^93^ It also fostered connections between Brazilian scholars and their counterparts in scientific centers, such as Germany and France. This carved out a place for Brazil in the global field of tropical medicine.^94^

However, as shown by the historian Simone Kropf, “Chagas disease was represented as a medico-scientific entity and at the same time as a social question.”^95^ Indeed, the 1910s-1920s were a period of intense discussion about the problems of Brazilian agriculture, coupled with the commemorations of the first independence centenary in 1922. In this context, Chagas disease was used by medical, political, intellectual elites as “the symbol of a ‘sick’ and ‘backwards’ country, devastated by endemics that disable the rural population, but its discovery was also seen “as the herald of [a new] science”.^96^ Therefore, a strong association was formed among the Brazilian elites, which framed Chagas disease as “a disease of Brazil.”^97^ Its presence in the territory was understood as an obstacle to the modernization of the country, which could be overcome through vector-destruction, sanitation, and home-improvement. Thus, the emergence of Chagas disease as a new pathology was intertwined with nation building and modernization projects in Brazil. Therefore, the history of Chagas disease in Brazil, like sleeping sickness in Africa, shows how disease emergence can be a matter of the scientific tools available to a society, and a result of political and social forces.^98^

## Conclusion

In conclusion, we would like to emphasize some challenges that humanities scholars face in studying the concept of emerging infectious diseases. Firstly, there are several gaps in the historiography of the EID concept, such as its circulation outside the USA and Western Europe, how scientific and political communities in the rest of the world have utilized it, and the evolution of the concept. There is considerable scope for future research into EIDs in global and non-western frameworks, particularly for historians working with multiple archives across various regions.

Secondly, it is worth reflecting on the utility of using techniques such as phylogenetics in tracing the ontological emergence of diseases in the past. On the one hand, they are vulnerable to charges of presentism and problematic in that they take modern science as a series of timeless facts that can be projected onto eras in which such knowledge never existed. On the other hand, when cognizant of this limitation, scientific methods, such as genetic studies, can provide valuable tools for writing histories of the emergence and persistence of the pathogens themselves. They allow historians to demonstrate the impacts of these nonhumans on multispecies demographies and geographies in periods where written records are scarce. Likewise, they can provide counterpoints to the prejudices of colonial archives.

Thirdly, although animals have not escaped the attention of historians of EIDs, there is still much more work to be done on animals as historical subjects in analyses of emerging or re-emergent diseases. Animals have played an important role in the microbiological reframing of old diseases, such as plague.^99^ Moreover, they are commonly blamed for the emergence of new diseases,^100^ notably avian influenza,^101^ HIV,^102^ and more recently, COVID-19. Yet, even in studies that explicitly focus on zoonotic transfers of disease, animal subjects often recede into the background and are cast as tools for human experimentation, vectors of infection, or passive victims of disease control policies, rather than as active beings that have shaped the emergence of diseases, and the knowledge produced about them. In taking animals seriously as historical subjects, one rich topic of enquiry would be to reverse the standard story of emerging infectious diseases and examine EIDs as a problem of wild animals. In addition to examining how contact with wild animals leads to spillovers of disease into human populations, we might also examine how diseases of humans or domestic animals spill into wild animal populations. Here, scholars could examine the attempts of scientists to protect mountain gorillas from human diseases, African wild dogs from diseases of domestic dogs, or even zoo and farm animals from COVID-19. The relationship between the global movements of animals and disease emergence constitutes another potential area of study. Along with the better-known stories of accidental introductions of rats and insects around the globe, historians could examine the epidemiological consequences of the trade in horses and cattle or wild animals for zoos, the migration of wild animals beyond international borders, or of sea creatures across the oceans.

A final challenge is how to transcend national/local boundaries when a analyzing the epistemological emergence of diseases. While some work has been done here regarding the history of plague, it is still lacking in other areas such as the study of trypanosomiasis. Despite the richness of the works mentioned above, they often regard the framing of trypanosomiasis as new entities as the result of processes sealed within national/imperial borders. However, Chagas disease, nagana, and sleeping sickness emerged almost in parallel, perhaps because both regions were exposed to similar global political, economic, and scientific processes. Thus, future works could highlight the connections between African and South American contexts, along with other places where trypanosomiases were studied, such as India or the Philippines. In short, global histories of disease emergence as an epistemological and social process constitute a promising field that can be developed beyond the cases discussed in this essay.

These are just a few suggestions that can be adapted to the study of the numerous infectious diseases not mentioned here. We have not aimed to be exhaustive, as this might have encompassed much of the history of infectious disease. Moreover, we did not examine the ontological and epistemological emergences of non-infectious diseases, nor how these diseases were examined or neglected by the EID concept. Despite these gaps, we hope our essay has provided a guide to works that have engaged critically with the EID concept, along with studies that, in connection with the burgeoning interest in this concept, have sought to understand the history of the ontological and epistemological emergence of new infectious diseases across space and time.

## Footnotes

2 J. Andrew Mendelsohn, “From Eradication to Equilibrium: How Epidemics Became Complex after World War I.,” in C. Lawrence and G. Weisz (Eds.), *Greater than the Parts: Holism in Biomedicine, 1920 – 1950* (Oxford: Oxford Univ. Press, 1998), 303-331.

3 The term “emerging disease,”, on the other hand, was first employed in 1962 in a veterinary journal to describe Equine piroplasmosis amongst horses. Ndow, J. Radeino Ambe, and Oyewale Tomori, “Emerging Infectious Diseases: A Historical and Scientific Review.”n *Socio-Cultural Dimensions of Emerging Infectious Diseases in Africa: An Indigenous Response to Deadly Epidemics*, eds. Godfrey B Tangwa et al. (Cham: Springer, 2019), 31-40.

4 National Science and Technology Council Committee on International Science, Engineering, and Technology Working Group on Emerging and Re-Emerging Infectious Diseases, *Infectious Disease - A Global Health Threat* (Washington, D.C.: Executive Office of the President of the United States, 1995), 9; Priscilla Wald, *Contagious: Cultures, Carriers, and the Outbreak Narrative* (Durham and London: Duke University Press, 2008), 29–30; Peter Washer, *Emerging Infectious Diseases and Society* (New York: Palgrave Macmillan, 2010), xii; Marcos Cueto, *Saúde global: uma breve história* (Rio de Janeiro: Editora FIOCRUZ, 2015), 71–72.

5 Frank M. Snowden, “Emerging and Reemerging Diseases: A Historical Perspective,” *Immunological Reviews* 225 (2008): 10–11.

6 Ibid., 9.

7 Stephen S. Morse and Ann Schluederberg, “Emerging Viruses: The Evolution of Viruses and Viral Diseases,” *The Journal of Infectious Diseases* 162, no. 1 (1 July 1990): 1–7.

8 Stephen S. Morse, ed., *Emerging Viruses* (New York: Oxford University Press, 1993); Lorna Weir and Eric Mykhalovskiy, *Global Public Health Vigilance: Creating a World on Alert* (New York, NY: Routledge, 2012), 32–33.

9 National Science and Technology Council Committee on International Science, Engineering, and Technology Working Group on Emerging and Re-Emerging Infectious Diseases, *Infectious Disease - A Global Health Threat*.

10 David Satcher, “Emerging Infections: Getting Ahead of the Curve,” *Emerging Infectious Diseases* 1, no. 1 (March 1995): 1–6.

11 Washer, *Emerging Infectious Diseases and Society*, 1–2; Nicholas King, “The Scale Politics of Emerging Diseases,” *Osiris* 19 (2004): 64.

12 Stephen S. Morse, “Factors in the Emergence of Infectious Diseases,” *Emerging Infectious Diseases* 1, no. 1 (March 1995): 7 and 13.

13 Ibid., 9.

14 Laurie Garrett, *The Coming Plague: Newly Emerging Diseases in a World out of Balance* (New York: Penguin Books, 1995); Snowden, “Emerging and Reemerging Diseases,” 14.

15 Washer, *Emerging Infectious Diseases and Society*, xi, 15.

16 King, “The Scale Politics of Emerging Diseases,” 74; For anthrax see Susan Jones, *Death in a Small Package : A Short History of Anthrax* (Baltimore: Johns Hopkins University Press, 2010).

17 Weir and Mykhalovskiy, *Global Public Health Vigilance*, 48–51.

18 Andrew Lakoff, “Global Health Security and the Pathogenic Imaginary,” in *Dreamscapes of Modernity* (University of Chicago Press, 2015), 305.

19 King, “The Scale Politics of Emerging Diseases,” 76.

20 Lakoff, “Global Health Security and the Pathogenic Imaginary”; See also Andrew Lakoff, “Two Regimes of Global Health,” *Humanity: An International Journal of Human Rights, Humanitarianism, and Development* 1, no. 1 (2010): 59–79.

21 Snowden, 2008 cited in Pierre-Olivier Méthot and Bernardino Fantini, “Medicine and Ecology: Historical and Critical Perspectives on the Concept of ‘Emerging Disease,”’ *Archives Internationales d'Histoire Des Sciences* 64, no. 172–3 (2014): 223.

22 Garrett, *The Coming Plague*, 7.

23 Expedito J. A. Luna, “A emergência das doenças emergentes e as doenças infecciosas emergentes e reemergentes no Brasil,” *Revista Brasileira de Epidemiologia* 5, no. 3 (December 2002): 229–43; Katia Abarca et al., “Tifus de los matorrales, una enfermedad emergente en Chile,” *Revista Chilena de Infectología* 35, no. 6 (2018): 696–99; Jon Arrizabalaga, “El desafío de las enfermedades (re)emergentes, los límites de la respuesta biomédica y el nuevo paradigma de Salud Global,” *História, Ciências, Saúde-Manguinhos* 28, no. 1 (March 2021): 255–81.

24 John N. Nkengasong and Sofonias K. Tessema, “Africa Needs a New Public Health Order to Tackle Infectious Disease Threats,” *Cell* 183, no. 2 (15 October 2020): 296–300; Ernest Tambo, Emmanuel Chidiebere Ugwu, and Jeane Yonkeu Ngogang, “Need of Surveillance Response Systems to Combat Ebola Outbreaks and Other Emerging Infectious Diseases in African Countries,” *Infectious Diseases of Poverty* 3, no. 1 (5 August 2014): 29; Godfrey B. Tangwa et al., eds., *Socio-Cultural Dimensions of Emerging Infectious Diseases in Africa: An Indigenous Response to Deadly Epidemics* (Cham, Switzerland: Springer, 2019).

25 Eveline M. Bunge et al., “The Changing Epidemiology of Human Monkeypox—A Potential Threat? A Systematic Review,” *PLOS Neglected Tropical Diseases* 16, no. 2 (11 February 2022): e0010141, https://doi.org/10.1371/journal.pntd.0010141.

26 Biénne Huisman, “Africa: Interview - “Guys, I Think That's a New Variant” - De Oliveira Reflects On Two Years Chasing the Virus,” *AllAfrica*, 6 June 2022, https://allafrica.com/stories/202206060426.html; See also Kevin Brandt, “Phaahla Says Monkeypox Outbreak a Cause for Concern as SA Records 5th Case,” *Eyewitness News*, 19 August 2022, https://ewn.co.za/2022/08/19/phaahla-says-monkeypox-outbreak-a-cause-for-concern-as-sa-records-5th-case.

27 Mirko D. Grmek, “Le concept de maladie émergente,” *History and Philosophy of the Life Sciences* 15, no. 3 (1993): 281–96.

28 A. D. Cliff et al., *Infectious Diseases: Emergence and Re-Emergence: A Geographical Analysis*, Oxford Geographical and Environmental Studies (Oxford ; New York: Oxford University Press, 2009), 7.

29 Brian Dolan, “It Wasn't Supposed to Be a Coronavirus: The Quest for an Influenza A(H5N1)-Derived Vaccine and the Limits of Pandemic Preparedness,” *Centaurus* 62, no. 2 (1 May 2020): 331–43.

30 Arrizabalaga, “El desafío de las enfermedades (re)emergentes.” Others have taken a less explicitly critical approach and instead invoked the term “emerging diseases” simply to bring histories of diseases into conversation with contemporary studies of COVID-19 and other new diseases. See Mark Honigsbaum and Pierre-Olivier Méthot, “Introduction: Microbes, Networks, Knowledge—Disease Ecology and Emerging Infectious Diseases in Time of COVID-19,” *History and Philosophy of the Life Sciences* 42, no. 3 (23 June 2020): 28, Maurits Bastiaan Meerwijk, “Phantom Menace: Dengue and Yellow Fever in Asia,” *Bulletin of the History of Medicine* 94, no. 2 (2020): 215–43.

31 Weir and Mykhalovskiy, *Global Public Health Vigilance*, 36.

32 Snowden, “Emerging and Reemerging Diseases: A Historical Perspective,” 23.

33 Warwick Anderson, “Natural Histories of Infectious Disease: Ecological Vision in Twentieth-Century Biomedical Science,” *Osiris* 19 (2004): 60.

34 Méthot and Fantini, “Medicine and Ecology: Historical and Critical Perspectives on the Concept of ‘Emerging Disease,”’ 214.

35 Grmek, cited in Pierre-Olivier Méthot, “Introduction: Mirko Grmek's Investigative Pathway,” in *Pathological Realities: Essays on Disease, Experiments, and History*, by Mirko D. Grmek, ed. Pierre-Olivier Méthot, Forms of Living (New York: Fordham University Press, 2019), 16.

36 Honigsbaum and Méthot, “Introduction: Microbes, Networks, Knowledge—Disease Ecology and Emerging Infectious Diseases in Time of COVID-19,” 28.

37 Linda Nash, “Beyond Virgin Soils: Disease as Environmental History,” in *The Oxford Handbook of Environmental History*, ed. Andrew C. Isenberg (Oxford: Oxford University Press, 2014), 97.

38 Ibid., 94.

39 Ibid., 96-97.

40 Ibid.

41 Woods et al argue that animals are rarely regarded as subjects of historical analysis in their own right in the history of medicine. Where they do appear, they tend to be “blank pages onto which humans” write meaning (Benson, 2011 cited in Woods et al, p 1). A similar criticism is made by Duarte about Brazilian historiography: Regina Duarte, “História dos animais no Brasil: tradições culturais, historiografia e transformação,” *Historia Ambiental Latinoamericana y Caribena (HALAC) Revista de La Solcha* 9, no. 2 (13 December 2019). Some exceptions include, most famously, Timothy Mitchell, *Rule of Experts: Egypt*, *Techno-Politics*, *Modernity* (Berkeley: University of California Press, 2002); and more recently Clapperton Chakanetsa Mavhunga, *The Mobile Workshop: The Tsetse Fly and African Knowledge Production* (Cambridge, Mass.: MIT Press, 2018); and Gabriel Lopes and Luísa Reis-Castro, “A Vector in the (Re)Making: A History of Aedes Aegypti as Mosquitoes That Transmit Diseases in Brazil,” in *Framing Animals as Epidemic Villains*, ed. Christos Lynteris (Cham: Palgrave Macmillan, 2019), 147–76.

42 Anne Hardy, “Animals, Disease, and Man: Making Connections,” *Perspectives in Biology and Medicine* 46, no. 2 (2003): 200–215.

43 For example, Frédéric Keck, *Avian Reservoirs: Virus Hunters & Birdwatchers in Chinese Sentinels Posts*, Experimental Futures (Durham: Duke University Press, 2020); Lyle Fearnley, *Virulent Zones: Animal Disease and Global Health at China's Pandemic Epicenter*, Experimental Futures (Durham London: Duke University Press, 2020).

44 Karen Brown, *Mad Dogs and Meerkats: A History of Resurgent Rabies in Southern Africa* (Athens: Ohio University Press, 2011).

45 Sujit Sivasundaram, “The Human, the Animal and the Prehistory of COVID-19,” *Past & Present* 249, no. 1 (1 November 2020): 296.

46 Ibid., 315.

47 For one account of this concept see Weir and Mykhalovskiy, *Global Public Health Vigilance*, chap. 2.

48 Méthot and Fantini, “Medicine and Ecology: Historical and Critical Perspectives on the Concept of ‘Emerging Disease,”’ 226.

49 Weir and Mykhalovskiy, *Global Public Health Vigilance*, 29.

50 For Weir and Mykhalovskiy, the “emerging” is prioritized over the “nonemerging.” See: Weir and Mykhalovskiy, 39.

51 Ibid., 61.

52 Richard Parker, “Sexuality, Culture, and Power in HIV/AIDS Research,” *Annual Review of Anthropology* 30 (2001): 168–69.

53 Paul Farmer, “Social Inequalities and Emerging Infectious Diseases, *Emerging Infectious Diseases* 2, no. 4 (1996): 267.

54 Richard Parker, “The Global HIV/AIDS Pandemic, Structural Inequalities, and the Politics of International Health,” *American Journal of Public Health* 92, no. 3 (1 March 2002): 344–45.

55 Méthot and Fantini, “Medicine and Ecology: Historical and Critical Perspectives on the Concept of “Emerging Disease,”’ 224.

56 Ibid., 228.

57 Carlo Caduff, *The Pandemic Perhaps: Dramatic Events in a Public Culture of Danger* (Oakland, California: University of California Press, 2015), 78.

58 Ibid., 79.

59 Carlo Caduff, “Pandemic Prophecy, or How to Have Faith in Reason,” *Current Anthropology* 55, no. 3 (2014): 301.

60 Caduff, *The Pandemic Perhaps*, 81.

61 Wald, *Contagious*, 31.

62 Ibid., 2.

63 Ibid., 39.

64 Ibid., 27.

65 Morse, “Factors in the Emergence of Infectious Diseases,” 7.

66 See, for instance, William Hardy McNeill, “Patterns of Disease Emergence in History,” in *Emerging Viruses*, ed. Stephen S. Morse (New York: Oxford University Press, 1993), 29–36; David Satcher, “Emerging Infections: Getting Ahead of the Curve,” *Emerging Infectious Diseases* 1, no. 1 (March 1995): 1–6.

67 Monica H. Green, “Emerging Diseases, Re-Emerging Histories,” *Centaurus* 62, no. 2 (1 May 2020): 234–47.

68 For an introduction to this concept, see Joël Coste et al., eds., “Le concept de pathocénose de M.D. Grmek: une conceptualisation novatrice de l'histoire des maladies,” École Pratique des Hautes Études, Sciences Historiques et Philologiques 5, *Hautes études médiévales et modernes* 109 (Genève: Librairie Droz S.A, 2016); Jon Arrizabalaga, “At the Intersection of Medical Geography and Disease Ecology: Mirko Grmek, Jacques May and the Concept of Pathocenosis,” *History and Philosophy of the Life Sciences* 40, no. 4 (6 December 2018): 71.

69 Mirko D. Grmek, “Preliminaries for a Historical Study of Diseases,” in *Pathological Realities: Essays on Disease*, *Experiments*, *and History*, ed. Pierre-Olivier Méthot, Forms of Living (New York: Fordham University Press, 2019), 31–40.

70 Méthot, “Introduction: Mirko Grmek's Investigative Pathway,” 3.

71 Alfred W. Crosby, “Virgin Soil Epidemics as a Factor in the Aboriginal Depopulation in America,” *The William and Mary Quarterly* 33, no. 2 (1976): 289–99. https://doi.org/10.2307/1922166.

72 William Hardy McNeill, *Plagues and Peoples* (New York: Anchor Books, 1998).

73 McNeill, “Patterns of Disease Emergence in History.”

74 McNeill, *Plagues and Peoples*, 10.

75 Laurent Binet, *Civilizations* (Paris: Grasset, 2020).

76 Mark Harrison, *Contagion: How Commerce Has Spread Disease* (New Haven: Yale University Press, 2012).

77 Myron Echenberg, *Plague Ports: The Global Urban Impact of Bubonic Plague*, *1894-1901* (New York: NYU Press, 2010).

78 Lukas Engelmann and Christos Lynteris, *Sulphuric Utopias: A History of Maritime Fumigation*, Inside Technology (Cambridge, Massachusetts: The MIT Press, 2019).

79 John Iliffe, *The African AIDS Epidemic: A History* (Oxford: James Currey, 2006).

80 Jacques Pépin, *The Origins of AIDS* (Cambridge: Cambridge University Press, 2021), 1–8, quote on 3.

81 Françous Simon, “Preface,” in *The Histories of HIVs: The Emergence of the Multiple Viruses That Caused the AIDS Epidemics*, ed. William H Schneider (Athens, Ohio: Ohio University Press, 2021), Preface (eBook).

82 William H Schneider, “The Histories of HIVs: The Multiple Viruses That Caused the AIDS Epidemic,” in *The Histories of HIVs: The Multiple Viruses That Caused the AIDS Epidemics*, ed. William H Schneider (Athens, Ohio: Ohio University Press, 2021), Chapter 1.

83 Ludwik Fleck, *Genesis and Development of a Scientific Fact*, trans. Thaddeus J. Trenn and Frederic Bradley (Chicago: University of Chicago Press, 1979); Charles E. Rosenberg, “Framing Disease: Illness, Society, and History,” in *Explaining Epidemics and Other Studies in the History of Medicine* (Cambridge: Cambridge University Press, 1992), 305–18.

84 Andrew Cunningham, “Transforming Plague: The Laboratory and the Identity of Infectious Diseases,” in *The Laboratory Revolution in Medicine*, ed. Andrew Cunningham and Perry Williams (Cambridge: Cambridge University Press, 1992), 234.

85 The history of plague microbiology beyond the identification of the bacillus and how it allowed a new framing of the disease has become richer and more global in the past decade. See, for instance, Deepak Kumar, ‘“Colony’ under a Microscope: The Medical Works of W.M. Haffkine,” *Science*, *Technology and Society* 4, no. 2 (September 1999): 239–71; Pratik Chakrabarti, *Bacteriology in British India: Laboratory Medicine and the Tropics* (Rochester: University of Rochester, 2012), 1–24; Christos Lynteris, “A ‘Suitable Soil’: Plague's Urban Breeding Grounds at the Dawn of the Third Pandemic,” *Medical History* 61, no. 3 (2017): 343–57; Matheus Alves Duarte da Silva, “From Bombay to Rio de Janeiro: The Circulation of Knowledge and the Establishment of the Manguinhos Laboratory, 1894–1902,” *História*, *Ciências*, *Saúde-Manguinhos* 26, no. 2 (2018): 1–19, Matheus Alves Duarte da Silva, “Quand la Peste Connectait le Monde : Production et Circulation de Savoirs Microbiologiques entre Brésil, Inde et France (1894–1922)” (Thèse de Doctorat, Paris, Ecole des Hautes Etudes en Sciences Sociales, 2020); Jules Skotnes Brown, “Pests, Knowledge and Boundaries in the Early Union of South Africa: Categorising, Controlling, Conserving” (Cambridge, University of Cambridge, 2020), chap. 4; Aro Velmet, *Pasteur's Empire: Bacteriology and Politics in France*, *Its Colonies and the World* (Oxford: Oxford University Press, 2020), chap. 1; Jules Skotnes-Brown, “Preventing Plague, Bringing Balance: Wildlife Protection as Public Health in the Interwar Union of South Africa,” *Bulletin of the History of Medicine* 95, no. 4 (2021): 464–96. In a different vein, historians have shown that the microbiological status of plague often cohabited with other non- biomedical perceptions, giving birth at times to hybrid understandings of plague. See, for instance, Projit Bihari Mukharji, *Nationalizing the Body: The Medical Market, Print and Daktari Medicine* (London: Anthem, 2011), chap. 5.

86 This should be treated as a gloss rather than a perfect translation. To translate this word before it passed into western medical vocabularies in 1895 would require a PhD length study. We are grateful to John Wright for this insight. “Andrew Koopman” [sic] (Adrian Koopman), cited in Lauren E.-A. Eyssen et al., “Recombinant and Native TviCATL from Trypanosoma Vivax: Enzymatic Characterisation and Evaluation as a Diagnostic Target for Animal African Trypanosomosis,” *Molecular and Biochemical Parasitology* 223 (1 July 2018): 50.

87 Histories of African trypanosomiasis and colonialism include: Maryinez Lyons, *The Colonial Disease: A Social History of Sleeping Sickness in Northern Zaire*, *1900–1940*, Cambridge Studies in the History of Medicine (Cambridge: Cambridge University Press, 1992); Helen Tilley, *Africa as a Living Laboratory: Empire, Development, and the Problem of Scientific Knowledge, 1870-1950* (Chicago: University of Chicago Press, 2011); Clapperton Mavhunga, *The Mobile Workshop: The Tsetse Fly and African Knowledge Production* (Cambridge, Mass.: MIT Press, 2018); Sarah Ehlers, *Europa und die Schlafkrankheit: Koloniale Seuchenbekämpfung, Europäische Identitäten und Moderne Medizin 1890-1950*, Kritische Studien zur Geschichtswissenschaft, Band 232 (Göttingen: Vandenhoeck & Ruprecht, 2019); Mari K. Webel, *The Politics of Disease Control: Sleeping Sickness in Eastern Africa, 1890-1920*, New African Histories (Athens, Ohio: Ohio University Press, 2019).

88 Karen Brown, “From Ubombo to Mkhuzi: Disease, Colonial Science, and the Control of Nagana (Livestock Trypanosomosis) in Zululand, South Africa, c. 1894–1953,” *Journal of the History of Medicine and Allied Sciences* 63, no. 3 (1 July 2008): 285–322; Daniel R Headrick, “Sleeping Sickness Epidemics and Colonial Responses in East and Central Africa, 1900-1940,” *PLoS Neglected Tropical Diseases* 8, no. 4 (24 April 2014): e2772–e2772.

89 John Ford, *The Role of the Trypanosomiases in African Ecology: A Study of the Tsetse Fly Problem* (Oxford: Oxford University Press, 1971).

90 Lyons, *The Colonial Disease: A Social History of Sleeping Sickness in Northern Zaire*, *1900–1940*.

91 Kirk Arden Hoppe, “Lords of the Fly: Colonial Visions and Revisions of African Sleeping-Sickness Environments on Ugandan Lake Victoria, 1906-61,” *Africa: Journal of the International African Institute* 67, no. 1 (1997): 86–105; Shirley Brooks, “Changing Nature: A Critical Historical Geography of the Umfolozi and Hluhluwe Game Reserves, Zululand, 1887 to 1947” (PhD Thesis, Kingston, Ontario, Queen's University, 2001); Brown, “From Ubombo to Mkhuzi: Disease, Colonial Science, and the Control of Nagana (Livestock Trypanosomosis) in Zululand, South Africa, c. 1894–1953”; Headrick, “Sleeping Sickness Epidemics and Colonial Responses in East and Central Africa, 1900-1940”; Mavhunga, *The Mobile Workshop: The Tsetse Fly and African Knowledge Production*.

92 Skotnes Brown, “Pests, Knowledge and Boundaries in the Early Union of South Africa: Categorising, Controlling, Conserving,” chap. 2.

93 Nancy Stepan, *Beginnings of Brazilian Science: Oswaldo Cruz, Medical Research and Policy*, *1890-1920* (New York: Science History Publications, 1976); Jaime Benchimol, *Manguinhos do sonho à vida: a ciência na Belle Époque* (Rio de Janeiro: Casa de Oswaldo Cruz, 1990); Jaime Benchimol and Luiz Antonio Teixeira, *Cobras*, *lagartos e outros bichos: uma história comparada dos institutos Oswaldo Cruz e Butantan* (Rio de Janeiro: Editora da UFRJ/Fundação Oswaldo Cruz, 1993); Jaime Benchimol, *Dos micróbios aos mosquitos: febre amarela e a revolução pasteuriana no Brasil* (Rio de Janeiro: Editora FIOCRUZ/Editora UFRJ, 1999); Henrique Cukierman, *Yes, nós temos Pasteur: Manguinhos, Oswaldo Cruz e a história da ciência no Brasil* (Rio de Janeiro: Relume-Dumará/FAPESP, 2007).

94 Simone Petraglia Kropf and Magali Romero Sá, “The Discovery of Trypanosoma Cruzi and Chagas Disease (1908-1909): Tropical Medicine in Brazil,” *História*, *Ciências*, *Saúde-Manguinhos* 16, no. suppl 1 (July 2009): 13–34.

95 Simone Kropf, *Doença de Chagas*, *doença do Brasil: ciência*, *saúde e nação*, *1909-1962* (Rio de Janeiro: Editora FIOCRUZ, 2009), 131.

96 Ibid., 131.

97 Chagas disease is not exclusively endemic to Brazil and is considered endemic in other Latin-American countries too. More recently, because of migration to Europe and North America, Chagas disease has been diagnosed in individuals living outside its endemic area. See Pablo Kreimer and Juan Pablo Zabala, “Chagas Disease in Argentina: Reciprocal Construction of Social and Scientific Problems,” *Science*, *Technology and Society* 12, no. 1 (March 2007): 49–72; Juan Pablo Zabala, *La enfermedad de Chagas en la Argentina: investigación científica*, *problemas sociales y políticas sanitarias* (Bernal, Buenos Aires: Universidad Nacional de Quilmes Editorial, 2010); Marina Gold, “Health, Migration, and NTDs: An Anthropological View,” *Medical Anthropology* 40, no. 6 (18 August 2021): 491–96.

98 Kropf, *Doenca de Chagas*, *doença do Brasil*, 131.

99 Frédérique Audoin-Rouzeau, *Les chemins de la peste: le rat*, *la puce et l'homme* (Rennes: Presses Universitaires de Rennes, 2003); Michael Vann, “Of Rats, Rice, and Race: The Great Hanoi Rat Massacre, an Episode in French Colonial History,” *French Colonial History* 4, no. 1 (2003): 191–203; Christos Lynteris, “Tarbagan's Winter Lair: Framing Drivers of Plague Persistence in Inner Asia,” in *Framing Animals as Epidemic Villains*, ed. Christos Lynteris (Cham: Springer International Publishing, 2019), 65–90.

100 Christos Lynteris, “Introduction: Infectious Animals and Epidemic Blame,” in *Framing Animals as Epidemic Villains* (Cham: Palgrave Macmillan, 2019), 2.

101 Keck, *Avian Reservoirs*.

102 Tamara Giles-Vernick and Stephanie Rupp, “From Chimps to Humans: A Historical Epidemiology of HIV Beginnings,” in *The Histories of HIVs: The Multiple Viruses That Caused the AIDS Epidemics*, ed. William H Schneider (Athens, Ohio: Ohio University Press, 2021), Chapter 4.

